# Predicting Late‐onset Alzheimer Disease (LOAD) following COVID‐19 infection by using machine learning applications on real world data from the National Clinical Cohort Collaborative (N3C)

**DOI:** 10.1002/alz70860_101746

**Published:** 2025-12-23

**Authors:** Carly Rose, William S Bush, Dana C. Crawford

**Affiliations:** ^1^ Case Western Reserve University, Cleveland, OH, USA; ^2^ Department of Population and Quantitative Health Sciences, Institute for Computational Biology, Case Western Reserve University, Cleveland, OH, USA; ^3^ Department of Population and Quantitative Health Sciences, Case Western Reserve University, Cleveland, OH, USA

## Abstract

**Background:**

The emergence of Coronavirus disease 2019 (COVID‐19) as a global pandemic is a new potential risk factor for LOAD that warrants investigation. To enhance the understanding of LOAD and its relationship with COVID‐19 among African Americans, we have leveraged clinical data from electronic health records (EHRs) available through the N3C.

**Methods:**

The N3C offers a unique opportunity to access national, de‐identified, individual‐level EHR data from nearly 100 institutions across the United States for rapid and meaningful research on COVID‐19. Using level 2 de‐identified EHR data from the N3C, we have defined a retrospective cohort to examine possible associations between COVID‐19 infection and LOAD diagnosis. From an initial population of nearly 24 million patients, we applied the following inclusion criteria: at least one clinical visit during the defined baseline period (one year before COVID was first diagnosed in the United States (1/20/2019‐1/20/2020)), no AD diagnosis at baseline, age 65, and no mention of “early” onset in the clinical record. This yielded a final cohort of approximately 3 million patients. Of these, ∼67,000 had been diagnosed with AD after the baseline period with a mean age of 77.8, was 57.4% female, and 13.4% Black or African Americans, compared to a mean age of 73.8, with 53.9% female, and 11.4% Black or African American for those without a LOAD diagnosis captured in the EHR.

**Results:**

Preliminary analyses consisted of univariate and multivariate logistic regression models, with LOAD as the outcome and COVID‐19 confirmed infection, sex and age as the predictor variables, and stratified by race. Results revealed impacts of COVID‐19 on LOAD risk across racial groups when adjusted for age and sex, with Black or African American patients showing increased risk of LOAD diagnosis following COVID‐19 infection (RR=1.07, 95% CI=(1.02‐1.11), *p*‐value=0.004) compared to white patients (RR=0.97, 95% CI=(0.95‐0.99), *p*‐value=0.001).

**Conclusions:**

Work is ongoing to develop random forest, neural network, and gradient boosting models to capture relationships and interactions logistic regression might miss, analyze and compare LOAD prediction accuracy across the models, and select additional features based on their importance and relative contributions to a LOAD diagnosis following COVID‐19 infection.

